# Is Crocin a Potential Anti-tumor Candidate Targeting Microtubules? Computational Insights From Molecular Docking and Dynamics Simulations

**DOI:** 10.3389/fmolb.2020.586970

**Published:** 2020-11-05

**Authors:** Ze Wang, Juan Ren, Nengzhi Jin, Xingyi Liu, Xiaofei Li

**Affiliations:** ^1^Department of Pharmaceutical Sciences, Zunyi Medical University at Zhuhai Campus, Zhuhai, China; ^2^Gansu Computing Center, Lanzhou, China; ^3^Center for Systems Biology, Department of Bioinformatics, School of Biology and Basic Medical Sciences, Soochow University, Suzhou, China

**Keywords:** tubulin, anti-tumor activity, molecular docking, molecular dynamics simulation, binding free energy, residue interaction network

## Abstract

Although it is known crocin, a hydrophilic compound from the herbal plant *Crocus sativus* L., has promising antitumor activity, the detailed mechanism of its antitumor activity was not well understood. Recent experiments suggested tubulin as the primary target for the antitumor activity of crocin. However, due to a lack of crystal structure of tubulin bound with crocin, the exact binding mode and interaction between crocin and tubulin remains exclusive. In the present work, a computational study by integrating multiple conformation docking, molecular dynamics simulation as well as residue interaction network analysis was performed to investigate the molecular mechanism of crocin-tubulin interaction. By comparing the docking score, the most likely binding mode CRO_E1 were identified from 20 different binding modes of crocin in the vinca binding pockets. Further molecular dynamics simulation of CRO_E1 complex showed the binding of crocin is more stable than the inhibitor soblidotin and vinblastine. During the simulation course, an excessive number of hydrogen bonds were observed for the ligand crocin. The binding free energy of crocin-tubulin complex was calculated as −79.25 ± 7.24 kcal/mol, which is almost twice of the ligand soblidotin and vinblastine. By using energy decomposition, hot residues for CRO_E1 were identified as Gln^11^, Gln^15^, Thr^72^, Ser^75^, Pro^173^-Lys^174^-Val^175^-Ser^176^-Asp^177^, Tyr^222^, and Asn^226^ in the β-chain, and Asp^245^, Ala^247^-Leu^248^, Val^250^, Asn^329^, and Ile^332^ in the α-chain. Residue interaction network analysis also showed the importance of these hot residues in the interaction network of crocin-tubulin complex. In addition, a common residue motif Val^175^-Xxx^176^-Asp^177^ was discovered for all three bindings, suggesting its importance in future drug design. The study could provide valuable insights into the interaction between crocin and tubulin, and give suggestive clues for further experimental studies.

## Introduction

Exploiting drug candidates from traditional Chinese medicine is of great interests in drug discovery. Saffron is the dried stigma of *Crocus sativus* L., which is a species of the Iridaceae family widely cultivated in China, Iran, India, Italy, Israel, Spain, and Turkey (Bathaie and Mousavi, 2010; Alavizadeh and Hosseinzadeh, 2014). Since ancient times, saffron is used as a dietary ingredient as well as medicinal herb in the treatment of various diseases (Bathaie and Mousavi, 2010). Crocin (CRO) is a hydrophilic carotenoid that are separated from saffron (Alavizadeh and Hosseinzadeh, 2014). As one of the main characteristic ingredients, CRO and its derivatives account for nearly 10% of total compounds in saffron (Pfander and Wittwer, 1975; Tsimidou and Tsatsaroni, 1993). Chemically, CRO is a di-glycosyl polyene ester of crocetin containing a 20-carbon carotenoid backbone and two D-gentiobioses as carbohydrate moieties (Alavizadeh and Hosseinzadeh, 2014). Experiments have shown that CRO has wide pharmacological effects including antioxidant, neuroprotective, antidepressant and antiproliferative (Alavizadeh and Hosseinzadeh, 2014). More importantly, the good hydrophilic property of CRO made it an attractive candidate in drug development.

Pharmacological studies showed that CRO exhibits promising antitumor activities (Bolhassani et al., 2014; Hoshyar and Mollaei, 2017). Several mechanisms were proposed to understand the antitumor activity of CRO, including inhibition of DNA and RNA synthesis (Abdullaev et al., 2003), interaction with topoisomerases (Bajbouj et al., 2012), induction of apoptosis (Sun et al., 2013; Amin et al., 2015), and so on. However, one of the drawbacks of these mechanisms is the lack of clarifying the primary target protein of CRO. Recently, biochemical as well as proteomic approaches suggested microtubules as the primary target of CRO (Hosseinzadeh et al., 2013; Hire et al., 2017; Sawant et al., 2019). Microtubule is a dynamic biopolymer composed of tubulin, which is a heterodimer composed of β and α subunit (Dumontet and Jordan, 2010). Microtubule dynamics, i.e., the assembly or disassembly of tubulin, plays essential roles in cell cycle (Dumontet and Jordan, 2010). The interference of microtubule dynamics could induce mitotic arrest and cell apoptosis. Due to the reason, tubulin is a target for a number of antitumor drugs including vinblastine (VBL), paclitaxel and colchicine (Dumontet and Jordan, 2010). It was found that CRO could competitively bind with tubulin at VBL site, disrupting microtubule dynamics and inhibiting cell proliferation (Hire et al., 2017; Sawant et al., 2019).

However, due to a lack of crystal structure of tubulin bound with CRO, the binding mode and detailed molecular interaction between tubulin and CRO is still unknown. In this work, we investigated the interaction between tubulin and CRO through computational approaches. The possible binding modes of CRO were explored through multiple conformation docking strategy. Then, molecular dynamics simulation was performed to fully consider the flexibility of tubulin and CRO. Molecular mechanics/generalized born surface area (MM/GBSA) method was applied to obtain a detailed energy contribution from key contact residues. Additionally, the underlying characteristics of key residues were analyzed from residue interaction network. Our study could provide valuable insights into the interaction between CRO and tubulin at molecular level, and give suggestive clues for further experimental studies.

## Materials and Methods

### Structure Preparation

The structure of tubulin having different vinca binding pockets were obtained from the Research Collaboration for Structural Bioinformatics protein database, including 1Z2B (bound with VBL), 3DU7 (bound with phomopsin A), 3E22 (bound with soblidotin, SBD) and 5NJH (bound with triazolopyrimidine). Molecular Operating Environment (MOE, 2019) software was used for structural preparation. Each structural data was cleaned by removing all unnecessary subunits and small molecules, leaving a ligand molecule, β and α-subunit. Missing amino acid residues and hydrogen atoms were added by QuickPrep in MOE. Energy minimization was performed by using Amber10 force field, with 0.1 RMS kcal/mol/A^2^ as a gradient.

### Multiple Conformation Docking With MOE

After the preparation of tubulin dimer with different vinca pocket conformations, multiple conformation docking was performed with MOE. In the multiple conformation docking strategy, an ensemble of different pocket conformations was used instead of a specific pocket conformation. Multiple conformation docking is different from traditional docking protocol, allowing the investigation and comparison of conformational variations of binding pockets. In order to compare the binding mode, all prepared tubulin structures were superimposed with reference to 1Z2B. The conformational difference of the binding pockets was measured by an MOE SVL script.

**FIGURE 1:**
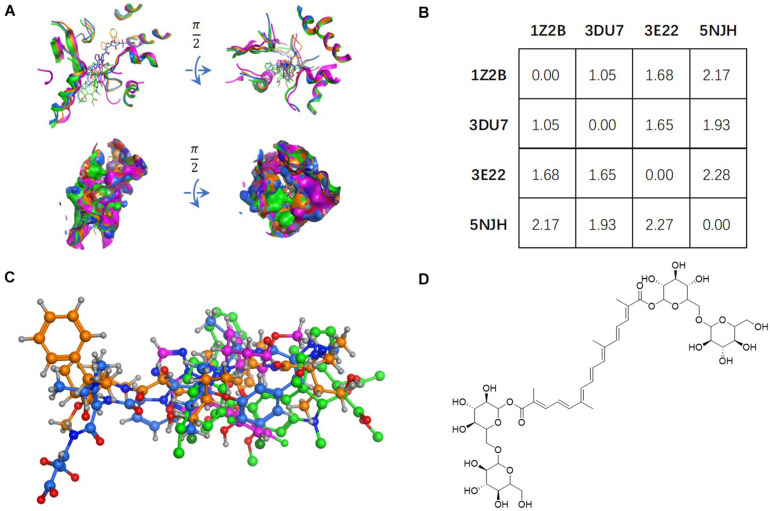
Tubulin bound with four different ligands (1Z2B, 3DU7, 3E22, and 5NJH, see the “Materials and Methods” part for more information) were compared by: **(A)** superposition of four binding pockets; **(B)** calculating RMSD matrix in angstrom; **(C)** superposition of four ligands. The chemical structure of CRO was shown in panel **(D)**.

Retrieved from PubChem, the ligand structure of CRO (Figure 1D) was imported in MOE and docked into the vinca binding site of each prepared conformation. The vinca binding site was defined as residues within 4.5 Å to the ligand of each prepared tubulin structure. For ligand docking with each receptor conformation, a set of 30 ligand conformations was produced to account for ligand flexibility. Docking structures were then refined by Amber10 force field and finally five poses were generated and ranked according to GBVI/WSA Δ*G* scoring method. The scoring function is defined as following:

(1) $$\Delta \,G_{b\,i\,n\,d}\approx \gamma \,\left[\frac{2}{3}\,\left(\Delta \,E_{e\,l\,e}+\Delta \,E_{s\,o\,l}\right)+\Delta \,E_{v\,d\,W}+\delta \,\Delta \,S\,A\right]+K$$

where Δ*E*_*ele*_, Δ*E*_*sol*_ and Δ*E*_*vdW*_ are the electrostatic, solvation, and van der Waals terms, respectively, Δ*SA* is exposed SA, *K* is the average entropy change, γ and δ are two parameters obtained by training. The GBVI/WSA Δ*G* scoring function was trained by the forcefield MMFF94x and AMBER99 to estimate the binding free energy between a ligand conformer and a binding pocket (Naïm et al., 2007). The CRO poses at each corresponding tubulin were filtered to remain the one with the highest Δ*G* score. After that, the CRO-tubulin complex was exported for further molecular dynamics simulations.

### Molecular Dynamics Simulations

After the docking step, a set of CRO binding modes were obtained for different pocket conformations of tubulin. Each binding complex was further analyzed by molecular dynamics (MD) simulation. The AMBER18 program was used to perform MD simulation. The ff14SB force field parameters were assigned to the prepared tubulin structure. For ligand molecules, the force field parameters described by General Force Field (GAFF) (Wang et al., 2004) were generated using the Antechamber program in AMBER18. The RESP charge fitting technique (Bayly et al., 1993; Cieplak et al., 1995; Fox and Kollman, 1998) was applied to calculate partial charges of ligands. The ligand and tubulin structure were then combined by using the LEaP program. A rectangular periodic box of water molecules was generated by using TIP3P water model (Jorgensen et al., 1983), extending at least 10 Å in each direction. The whole system was neutralized with sodium ions as counterions.

Three steps of minimization were performed in prior to MD simulation. In the first stage, only the positions of water molecules were optimized by fixing ligand-tubulin complex with a restraint force constant of 10.0 kcal/mol/Å^2^. In the second stage, the restrains on the complex were partially released by only fixing C_α_, N, O with a restraint constant of 5.0 kcal/mol/Å^2^. In the third stage, the entire system in solvated box was minimized by releasing all restraints. Each minimization steps contained 10,000 cycles including the first 1,000 cycles of the steepest descent algorithm and the remaining 9,000 cycles of conjugate gradient method. The minimized structure was used as starting input for MD simulation. The temperature of system was gradually raised from 0 to 300 K in 200 ps canonical ensemble (fixed N, V, and T) heating process by applying the Langevin dynamics with a collision frequency of 2.0. The system was equilibrated by 300 ps NPT equilibration (fixed N, P, and T) at 1.0 bar and 300 K, with all residues restrained by a force constant of 1.0 kcal/mol/Å^2^. Finally, the position restraints were released, and a production phase of 90 ns was performed under the same conditions as in NPT equilibration. Coordinates were saved for every 10 ps. In all of the MD simulations, 2.0 fs was used as time step and 8.0 Å was used as short-range cutoff value for non-bonded interactions. The long-range electrostatic interactions were calculated through the particle-mesh Ewald (PME) method (Darden et al., 1993). Bond restraints including hydrogen atoms were realized by applying SHAKE algorithm (Ryckaert et al., 1977). MD trajectories were processed and analyzed by evaluating RMSD value of the tubulin and ligands. The RMSF and the hydrogen bond analysis were performed by cpptraj tool in AMBER18. The same protocol was applied for all simulation processes of different conformation of binding pockets and ligands.

### MM/GBSA Binding Energy Calculation

For each ligand-tubulin complex, the MD trajectory was used to estimate the binding energy (Δ*G*_*total*_) between ligand and tubulin, which is the sum of van der Waals, electrostatic, polar and non-polar solvent energies. To effectively calculate the binding energy, MM/GBSA method (Wang et al., 2017, 2019) was applied to the following thermodynamic relation:

(2) $$\Delta \,G_{b\,i\,n\,d,s\,o\,l}=\Delta \,G_{b\,i\,n\,d,v\,a\,c}+\Delta \,G_{c\,o\,m,s\,o\,l}-\left(\Delta \,G_{l\,i\,g,s\,o\,l}+\Delta \,G_{r\,e\,c,s\,o\,l}\right)$$

where Δ*G_*bind*,__*sol*_* and Δ*G*_*bind, vac*_ are the binding energies in solvent condition and vacuum condition, respectively, and Δ*G*_*com, sol*_, Δ*G*_*lig, sol*_ and Δ*G*_*rec, sol*_ are the solvation free energies of complex, ligand, and receptor, respectively. The solvation free energy can be attributed to an electrostatic and a non-electrostatic contribution through the equation:

(3) $$\Delta \,G_{s\,o\,l}={G_{e\,l\,e}|}_{\epsilon =1}^{\epsilon =80}+\Delta \,G_{n\,o\,n\,e\,l\,e}$$

The electrostatic contribution can be solved by the linearized GB method, while the non-electrostatic contribution can be estimated by an empirical SA term. In this study, we used the solute dielectric constant of 1, the solvent dielectric constant of 80, and water probe radius of 1.4 Å. Δ*G*_*vac*_ is determined by calculating non-bonded interaction energy (Δ*E*_*MM*_) between ligand and receptor and entropy change (Δ*S*_*NMA*_) during ligand binding:

(4) $$\Delta \,G_{v\,a\,c}=\Delta \,E_{M\,M}-T∙\Delta \,S_{N\,M\,A}$$

In case of different ligands binding to the same protein, the entropy contribution can be neglected if only the hotspot residues and interaction features rather than the absolute Gibbs free energy were to be evaluated. For this reason, we collected multiple snapshots from MD trajectory for the MM/GBSA calculation at 100 ps intervals. The binding energies between different conformations of binding pocket of tubulin and ligands were obtained and compared for further analysis. In addition, to achieve a detailed picture of the interaction between ligand and tubulin, MM/GBSA method was applied to decompose the interaction energy at a per-residue basis without considering entropy contributions.

### Residue Network Calculation

The web server RING-2.0 (Piovesan et al., 2016) was used to build the residue interaction network by using protein and protein-ligand structures. RING-2.0 algorithm could derive a network through two steps, i.e., identifying node-node pair by measuring physical distance and recognizing the interaction type of each pair (Piovesan et al., 2016). In the computation, we have considered all atoms of each residue for distance measurement and display only one interaction per interaction type for simplicity reason. Then, the derived networks were imported into Cytoscape (Shannon et al., 2003) for topological analysis. In the network graph, residues and interactions between residues were represented as nodes and edges between nodes, respectively. The degree, betweenness and closeness centrality was computed by using NetworkAnalyzer (Assenov et al., 2007), which are key values measuring the importance or centrality of a node in the network.

## Results and Discussion

### Multiple Conformation Docking

The vinca binding pocket of tubulin dimer has different conformations while bound to different inhibitors. As screened from the Protein Data Bank, at least four entities were found to represent tubulin bound to structurally different inhibitors at vinca binding pocket. The PDB structures include tubulin-VBL complex (PDB ID: 1Z2B), tubulin-phomopsin A complex (PDB ID: 3DU7), tubulin-SBD complex (PDB ID: 3E22) and tubulin-triazolopyrimidine complex (PDB ID: 5NJH) (Figure 1C). According to induced-fit theory, the shape of the binding cavity will change according to ligand geometries. Comparison of these binding pockets indicated a great deal of structural variety upon binding of structurally diverse ligands (Figure 1A). The RMSD matrix showed the structural differences among four binding pockets. Despite the similarity between the binding pockets of 1Z2B and 3DU7, the binding cavities varies significantly (with RMSDs > 1.5 Å) (Figures 1A,B).

Ligand geometry could significantly change the conformation of the same binding pocket. Since the binding mode of CRO is largely unknown, the exploration of docking by different conformations of binding pockets allows to probe the binding mode and interaction between CRO and tubulin. In the study, CRO was docked into four different conformations of the binding pocket of tubulin by using MOE software. The selection of tubulin structures (PDB ID: 1Z2B, 3DU7, 3E22, and 5NJH) from the Protein Data Bank helps to investigate and compare different binding modes of CRO.

**FIGURE 2:**
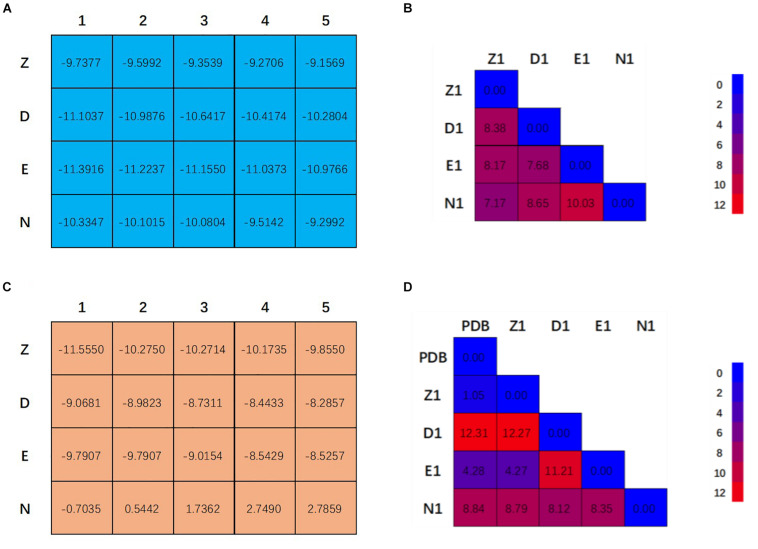
Twenty binding modes for CRO **(A)** and VBL **(C)** discovered by multiple conformation docking strategy. The RMSD matrix of CRO **(B)** and VBL **(D)** was created to compare the different ligand conformations. For VBL, PDB structure was used to demonstrate the possibility of screening correct ligand conformation by using multiple conformation docking strategy. All units are in Å.

**FIGURE 3:**
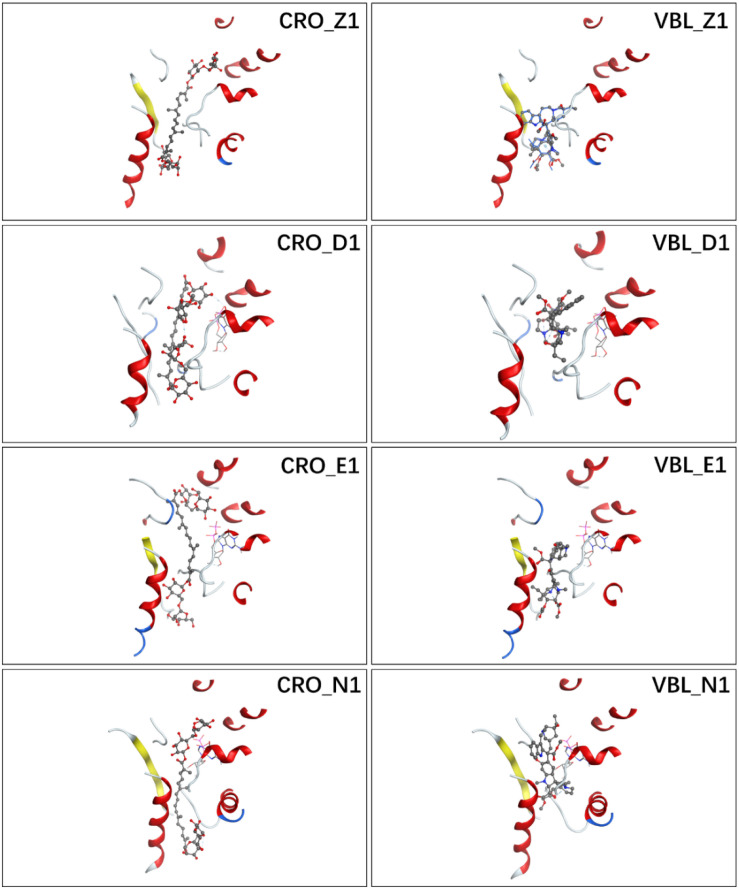
Ligand conformations with the highest docking scores of CRO **(left)** and VBL **(right)** in the binding pocket.

Figure 2A showed the docking matrix of possible binding modes of CRO by multiple conformation docking method. Each row represents the conformation of the binding pocket of tubulin, where Z, D, E, and N stands for 1Z2B, 3DU7, 3E22, and 5NJH, respectively. By MOE docking, the first five top ranked conformers of CRO were listed for each binding pocket. The binding matrix therefore has collected a total number of 20 different binding modes of CRO (Figure 2A). The RMSD values of the screened conformers of CRO were calculated and listed in Figure 2B. Ranging from 7.07 to 12.26 Å, the RMSD matrix indicated that a significantly diversity of the ligand geometry was obtained from the multiple conformation docking method. The conformers of CRO with the highest score in each binding pocket were shown in Figure 3. As shown in the figure, the geometry and orientation of CRO differs significantly in the four binding pockets. In fact, the conformers of VBL and their locations in four pockets also varies in different binding pockets (Figure 2C). This indicated the flexibility of ligand in binding with a specified pocket conformation, and also rationalized the necessity for performing multiple conformation docking.

The detailed interactions between tubulin and ligand for different binding modes were analyzed, and residues involving the binding interaction were plotted in Supplementary Figure S1. The interacting residues were highlighted in the protein sequence as shown in Supplementary Figure S2 (for ligand CRO) and S3 (for ligand VBL). The 2D map is a projection of 3D structure in Figure 3, which provides a clear representation of the binding interaction in 3D structure. Interestingly, although the ligand pose varies significantly (Figures 2B,D), some common modes were observed for the protein residues involving the binding interaction. For the ligand CRO, the common modes shared Gln^15^, Val^175^-Ser^176^, Tyr^208^, Pro^220^-Thr^221^-Tyr^222^ in the β-chain, and Leu^248^, Pro^325^, Val^328^-Asn^329^, Ile^332^, Phe^351^, Val^353^, Ile^355^ in the α-chain. Similar patterns were observed in the binding mode of the ligand VBL, including Val^175^-Ser^176^, Pro^220^-Thr^221^-Tyr^222^ in the β-chain, and Leu^248^, Pro^325^, Val^328^-Asn^329^, Phe^351^, Val^353^, Ile^355^ in the α-chain.

As can be seen in Figure 2A, E1 for CRO is the most favorable binding mode from the perspective of binding energy. Actually, the GBVI/WSA Δ*G* scores for the first three modes in 3E22 binding pocket are higher than other investigated binding pockets, indicating 3E22 is the most likely conformation for the binding pocket of CRO. In comparison, the most favorable binding mode for VBL is Z1. Since the crystal structure of tubulin bound to VBL has been solved, we compared the predicted binding mode Z1 with its crystal structure. As shown in Figure 2D, the RMSD value between Z1 and its PDB structure is 1.08 Å, meaning the computed binding mode is highly similar to its crystal structure. This suggests our method of multiple conformation docking is useful in finding the correct binding mode. For this reason, we will use the binding mode E1 for CRO as the starting structure for further investigation.

Ideally, the screening of the correct binding modes was achieved through calculating of some physical quantities, such as binding energy, by averaging over an infinite conformational space of both ligand and binding pocket. According to the ergodic hypothesis, this is equivalent to performing time average from zero to infinity (Cramer, 2004). In molecular dynamics simulation, a finite period of time (typically in nanosecond scale) was engaged to focus on the most representative microstates of an ensemble. Therefore, it is necessary to enumerate representative microstates of the CRO-tubulin complex.

### Molecular Dynamics Simulations

**FIGURE 4:**
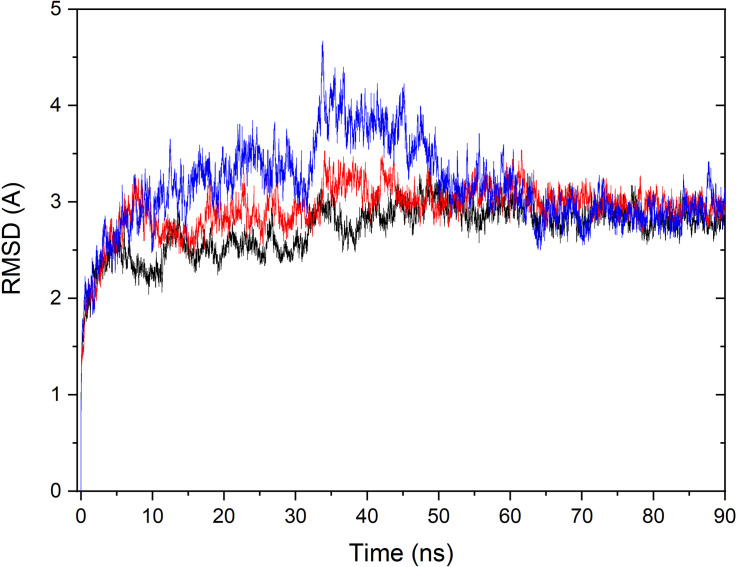
Monitoring of RMSD change over the MD simulation course for the tubulin bound with ligand CRO (black), VBL (blue), and SBD (red). The RMSD value of C_α_ of each MD trajectory was calculated and plotted against simulation time.

Based on the constructed structure of CRO-tubulin complex identified in the multiple conformation docking step, MD simulations were performed to further achieve rationalized and stable complex. The stability of tubulin and CRO in the binding site were assessed by the root-mean-square derivation (RMSD) values of C_α_ atoms with respect to the initial conformation during the MD simulation period, as shown in Figure 4. Since SBD is the ligand molecule in crystal structure of 3E22, MD trajectories of SBD and VBL were obtained and RMSD values of C_α_ atoms were plotted accordingly for comparison (Figure 4). Significant fluctuations in RMSD plots were observed in the first 60 ns for all three ligands CRO, SBD, and VBL, indicating protein domain movements upon ligand binding. Then the three RMSD curves achieved stable plateaus for the last 30 ns. In the stable stage, the RMSD values kept at around 2.8 Å with respect to the initial protein conformation. However, in the first 60 ns the RMSD fluctuations of CRO is significantly smaller than VBL and SBD. This means a slighter conformational change of tubulin upon CRO binding as compared to VBL and SBD. It is likely the ligand CRO is better accommodated in the protein than VBL and SBD.

**FIGURE 5:**
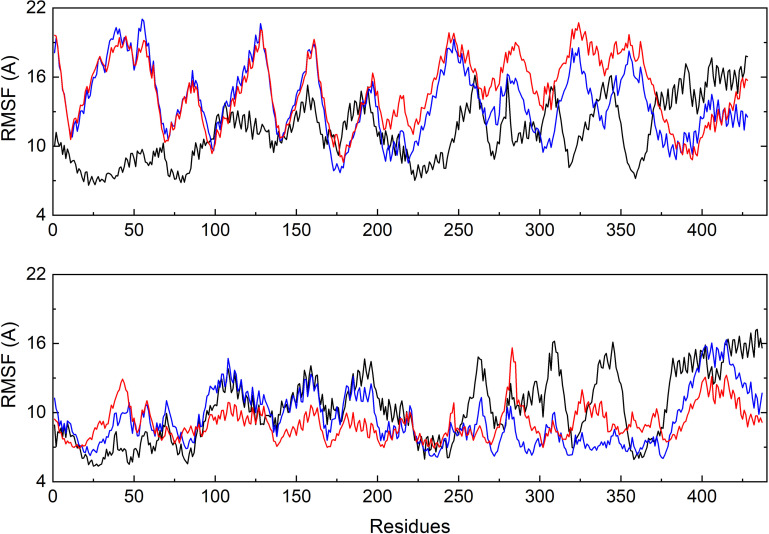
Comparison of the backbone RMSF values of tubulin bound with different ligands CRO (black), SBD (blue) and VBL (red). The β **(top)** and α **(below)** chain were plotted separately.

To further investigate the flexible protein segments attributing the RMSD fluctuations, the root-mean-square fluctuation (RMSF) values of tubulin upon binding of each ligand were calculated based on the all-atom MD trajectories (Figure 5). It could be discovered that the average fluctuations of CRO binding is smaller SBD and VBL. The RMSF curves of SBD and VBL are highly similar, but are significantly distinct from CRO. This suggests a different binding mode of CRO from the traditional inhibitors SBD and VBL. Furthermore, a lower average RMSF value throughout tubulin indicate the CRO binding mode is more favorable than SBD and VBL.

### Hydrogen Bond Analysis

**FIGURE 6:**
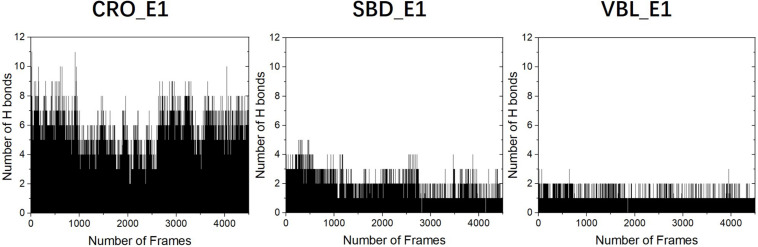
The analysis of hydrogen bonds between tubulin and different ligands CRO **(left)**, SBD **(middle)**, and VBL **(right)**. The density of frame was 50 frames/ns.

To primarily investigate the binding affinity between the ligands and tubulin, we performed hydrogen bond analysis along the 90 ns MD trajectories of each ligands. The results were presented in Figure 6. The frequencies of hydrogen bonding between tubulin and the ligand CRO, SBD, and VBL were plotted versus snapshots extracted from MD trajectories. As demonstrated in Figure 6, the average frequency of hydrogen bonding of CRO was around 6, which is larger than the average frequency of SBD and VBL. Although the strength of each hydrogen bond was not considered yet, but it is highly likely that the formation of excessive amounts of hydrogen bond between CRO and tubulin will lead to a much more stable binding mode than SBD and VBL. In the next part, the binding energy of each ligand will be further analyzed by MM/GBSA methodology.

### MM/GBSA Binding Energy Calculation

To estimate the binding energy of ligands and tubulin, MM/GBSA method was performed to calculate energy contributions (Wang et al., 2017, 2019). The three methodologies of MM, GB, and SA were utilized to compute energy contributions from van der Waals (vdw), electrostatic (ele), polar and non-polar surface solvation interactions (Wang et al., 2017, 2019). According to the all-atom MD trajectories shown in Figure 4, the last 20 ns frames were all considered to perform MM/GBSA for all three ligands. A total number of 2,000 frames were extracted for the computation to obtain reliable binding free energies. It should be pointed out that a complete estimation of binding free energy includes the calculation of entropy contribution. However, since we are interested in elucidating the dominate factors in different binding modes rather than computing the exact value of free energy, therefore the computationally expensive entropy calculations were neglected in this part.

The computed results of MM/GBSA and corresponding energy components terms for the three ligands were listed in Table 1. The methodology of MM/GBSA allows detailed decomposition of the free energy into different interaction contributions, which is convenient for the analysis of each term separately. As shown in Table 1, the polar solvation energies are the only unfavorable terms for all three ligands. And the remaining terms of the van der Waals, the electrostatic and the non-polar solvation interactions have attributed a total energy of −79.25 ± 7.24, −40.94 ± 3.71, and −42.49 ± 2.95 kcal/mol for the ligand CRO, SBD, and VBL, respectively. This means the binding of three ligands are thermodynamically favorable, which is accordance with experimental observations that all three ligands are good inhibitors for tubulin. On the other hand, the binding free energy of CRO is almost twice of traditional inhibitors SBD and VBL, suggesting its potential high inhibition efficiency toward tubulin.

**TABLE 1:** MM/GBSA binding energy between the protein and different ligand CRO, SBD and VBL.

Mode	Contribution (kcal/mol)				Δ*G*_*total*_ (kcal/mol)
	vdw	ele	Polar	Non-polar	
CRO_E1	–89.64	–115.30	138.94	–13.25	−79.25 ± 7.24
SBD_E1	–56.84	–381.00	404.62	–7.72	−40.94 ± 3.71
VBL_E1	–66.07	–39.94	71.87	–8.32	−42.49 ± 2.95

### Key Residues Analysis

**FIGURE 7:**
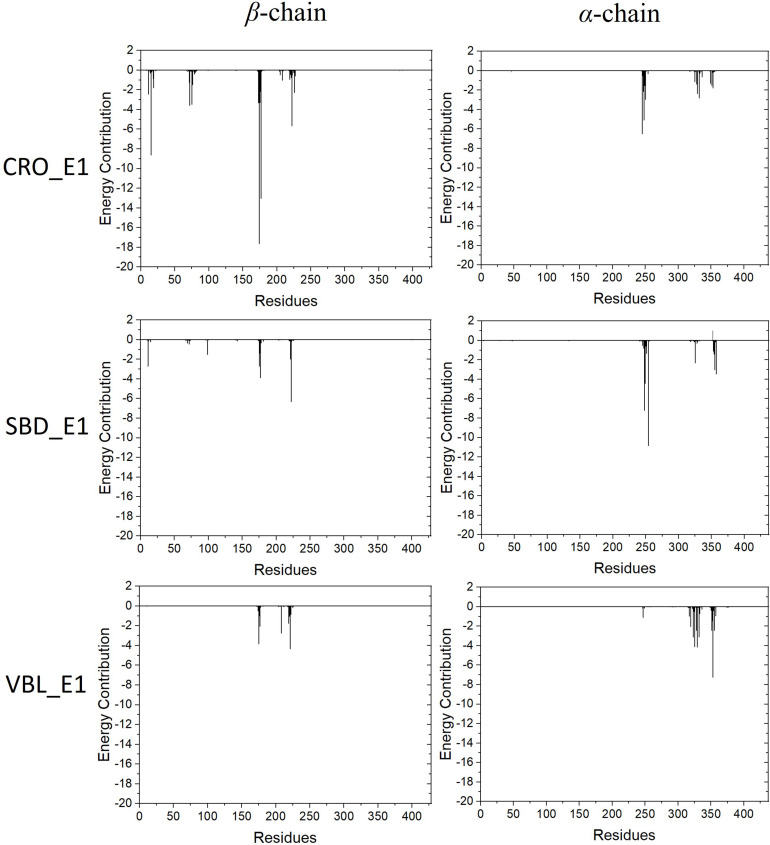
Energy contributions of each residue-ligand pair for the ligand CRO **(top)**, SBD **(middle)** and VBL **(bottom)**.

The energy contribution of each residue-ligand pair was decomposed to obtain a detailed energy analysis on the interaction between tubulin and the ligands. By using this quantitative analysis, it is helpful to investigate and identify key residues as hot spots involving in the binding interaction. The decomposed binding free energies of each residue-ligand pair were plotted versus the position number of each amino acid in Figure 7. The peaks in the figure showed the energy contributions of each residue.

**FIGURE 8:**
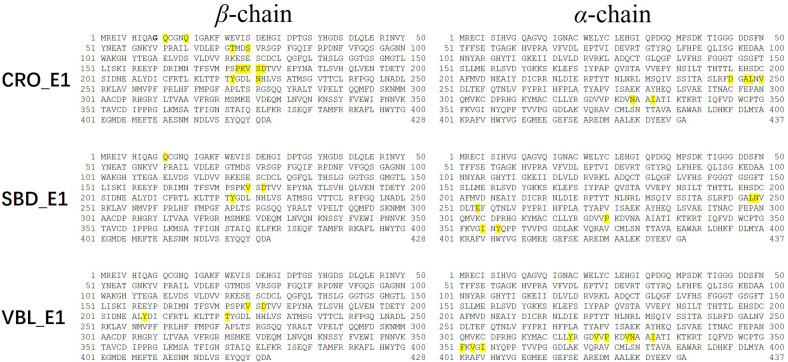
Key residues distribution in β and α chain for ligand CRO **(top)**, SBD **(middle)** and VBL **(bottom)** by MM/GBSA energy decomposition method.

Residues with an absolute energy contribution larger than 2 kcal/mol were identified as hot residues. For the CRO binding, 11 (Gln^11^, Gln^15^, Thr^72^, Ser^75^, Pro^173^-Lys^174^-Val^175^-Ser^176^-Asp^177^, Tyr^222^, and Asn^226^) and 6 (Asp^245^, Ala^247^-Leu^248^, Val^250^, Asn^329^, and Ile^332^) hot residues were identified in the β and α chain, respectively (Figure 8). In comparison, 4 (in β chain) and 6 (in α chain) hot residues were identified for SBD, and 4 (in β chain) and 9 (in α chain) were identified for VBL (Figure 8). Clearly, the total energy contributions of hot residues in the binding of CRO is larger than SBD and VBL. This is in line with energy analysis by MM/GBSA method, indicating a strong interaction between CRO and tubulin. In addition, the hot residues in the beta chain involving in the binding of CRO were significantly different from SBD and VBL, suggesting a distinct binding mode of CRO. An interesting binding motif of Val^175^-Xxx^176^-Asp^177^ in the beta chain was discovered to the common element involving the binding of different ligands CRO, SBD and VBL. This peptide motif may serve as a critical site for further development of tubulin inhibitor.

In order to compare the CRO-tubulin complex structure before and after molecular dynamics simulation, a snapshot at 80 ns in the stable plateau of MD trajectory of CRO_E1 was extracted as a representative structure of the post-MD structure. The 2D interaction map and pocket residues were shown in Supplementary Figure S4. It should be noted that the 2D interaction map in Supplementary Figure S4 is different from the hot residue map in Figure 8. The hot residue map considers the average energy contribution (>2 kcal/mol) throughout the MD simulation period, while the 2D interaction map identifies important pocket residues from a static structure. The comparison of 2D interaction map between pre-MD (Supplementary Figure S2) and post-MD (Supplementary Figure S4) showed the common residues were reserved, including Gln^15^, Val^175^-Ser^176^, Pro^220^-Thr^221^-Tyr^222^ in the β-chain, and Leu^248^, Pro^325^, Val^328^-Asn^329^, Ile^332^, Phe^351^, Val^353^ in the α-chain, which indicates the interacting residues in the binding pocket were conserved features for CRO.

### Community Network Between CRO and Tubulin

**FIGURE 9:**
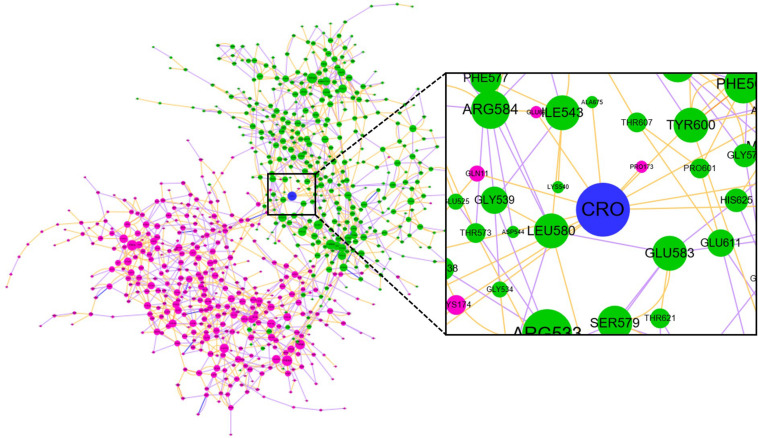
The residue interaction network of tubulin-CRO complex. Each node represents a residue in β chain (pink), α chain (green) or ligand (blue). The size of each node was linearly correlated with the degree of the node. The label of each node was numbered sequentially by taking the two chains as one sequence, which means the number 1–428 and 429–865 represents β and α chain, respectively. Each edge represents the interaction between nodes, including hydrogen bonding (purple), ionic interaction (blue), van der Waals interaction (yellow), and π–π stacking (orange).

Community network analysis of protein, also named as residue interaction network (RIN) analysis, is a valuable method in deciphering the topology and dynamics of protein structure (Shcherbinin et al., 2019). By modeling a protein structure as residue nodes and interaction edges, the RIN approach allows to uncover key characteristics of the protein as well as rationalize drug design by topologically measuring the binding interactions (Hu et al., 2014; Liang et al., 2018, 2019, 2020). To better understand the interaction between CRO and tubulin, the RIN of tubulin and its bound state with CRO were constructed accordingly (Figure 9). As shown in Figure 9, residues involving in more than one interaction with the remaining residues or ligand (blue node) were represented as nodes. Residues in the β and α chain of tubulin were colored in pink and green, respectively. In the RIN, the non-covalent interactions including hydrogen bonding (purple), ionic interaction (blue), van der Waals interaction (yellow), and π–π stacking (orange) were represented as undirected edges between nodes. For simplicity, only one interaction per interaction type was plotted in the network. The discussion here was based on the interaction simplified network, but it should be pointed out that the discussion could be extended to an advanced network with multiple edges between nodes.

In network graph theory, the degree, betweenness and closeness centrality are characteristic values for measuring the importance of a node in a network (Shcherbinin et al., 2019). To investigate the ligand-binding induced change of the key residues as discovered in molecular dynamics simulation, we have computed the degree, betweenness and closeness centrality of the ligand and key residues. A comparison of the degree, betweenness and closeness centrality of the key residues between tubulin and tubulin bound with CRO were listed in Table 2. It could be found that the degree, betweenness and closeness centrality of each node was increased after the binding of the ligand CRO. Actually, the betweenness and closeness centrality of CRO (0.3199 and 0.1481, respectively) were ranked the highest in the network, indicating its vital importance in the interaction with tubulin. Therefore, the key residues were deeply connected with other parts of the network through the interaction with CRO. In other words, their importance or centrality in the network was increased after binding with the ligand, supporting the conclusion from the previous MD analysis.

**TABLE 2:** Comparison of the degree, betweenness and closeness centrality of the key residues of tubulin and tubulin-CRO complex.

Chains	Residues	Tubulin-CRO complex			Tubulin		
		Betweenness	Closeness	Degree	Betweenness	Closeness	Degree
B	GLN11	0.0000	0.1307	2	0.0000	0.0775	1
B	THR72	0.0449	0.1331	5	0.0053	0.0839	4
B	PRO173	0.0000	0.1290	1	NA	NA	0
B	LYS174	0.0080	0.1293	3	0.0000	0.0841	2
B	VAL175	0.0945	0.1413	4	0.0013	0.1014	3
B	TYR222	0.1619	0.1417	5	0.0006	0.0968	4
C	ALA675	0.0000	0.1290	1	NA	NA	0
C	LEU676	0.0109	0.1302	3	0.0009	0.0881	2
C	VAL678	0.1983	0.1425	3	0.0039	0.1011	2
C	ASN757	0.0381	0.1339	4	0.0030	0.1011	3

*1) NA means the node was neglected by Cytoscape since its degree was deduced to 0; 2) the label of each node was numbered sequentially by taking the two chains as one sequence, which means the number 1–428 and 429–865 represents β and α chain, respectively.*

## Conclusion

Currently, the crystal structure of tubulin bound with CRO is still lacking, which hinders our understanding of the interaction between CRO and tubulin. In this paper, we have screened the most likely binding mode CRO_E1 of CRO in the vinca binding pocket of tubulin based on multiple conformation docking strategy. Furthermore, molecular dynamics simulation method was involved to investigate the mechanism of interaction of CRO_E1. The results showed the excessive number of hydrogen bonds of CRO_E1 plays an important role in the CRO-tubulin binding. Energic analysis showed the binding free energy of CRO is almost as twice as the inhibitor soblidotin and VBL, suggesting a favored binding of CRO in the vinca binding pocket of tubulin. Hot residues were analyzed by energy decomposition, and were shown to be in accordance with their topological characteristics in the interaction network. Although hot residues involving the binding were different, a common residue motif Val^175^-Xxx^176^-Asp^177^ was identified for the three ligands, suggesting its importance in future drug design. The results in this paper provide new insights into structural basis of the interaction between CRO and tubulin, which is valuable for future drug design and development targeting tubulin.

## Data Availability Statement

The raw data supporting the conclusions of this article will be made available by the authors, without undue reservation.

## Author Contributions

ZW conceptualized the methodology. ZW, JR, and NJ performed the MD simulations. ZW and XLn performed residue interaction network analysis. ZW and XLa analyzed the data. ZW and JR wrote the manuscript. All authors contributed to the article and approved the submitted version.

## Conflict of Interest

The authors declare that the research was conducted in the absence of any commercial or financial relationships that could be construed as a potential conflict of interest.
