# Supplementary material for: Is Crocin a Potential Anti-tumor Candidate Targeting Microtubules? Computational Insights From Molecular Docking and Dynamics Simulations
Source: Front Mol Biosci. 2020 Nov 5;7:586970. doi: 10.3389/fmolb.2020.586970 (PMC7674667; doi:10.3389/fmolb.2020.586970)
Supplement: Supplementary file 1 [file Data_Sheet_1.PDF]

Supplementary Information

Is Crocin a Potential Anti-tumor Candidate Targeting Microtubules? Computational Insights from Molecular Docking and Dynamics Simulations

Ze Wang[1†*], Juan Ren[1†], Nengzhi Jin[2], Xingyi Liu[3], Xiaofei Li[1*]

1 Department of Pharmaceutical Sciences, Zunyi Medical University at Zhuhai Campus, Zhuhai, PR China

2 Gansu Computing Center, Lanzhou, PR China

3 Center for Systems Biology, Department of Bioinformatics, School of Biology and Basic Medical Sciences, Soochow University, Suzhou, PR China

† These authors have contributed equally to this work

* corresponding author

Email Addresses: wz@zmu.edu.cn (Z. Wang), lixiaofei@zmu.edu.cn (X. Li)

Figure Captions

Figure S1. Detailed interactions between tubulin and the ligand CRO (left) and VBL (right) with the highest docking scores.

Figure S2. Residues (highlighted in yellow) involving the binding interaction with the ligand CRO.

Figure S3. Residues (highlighted in yellow) involving the binding interaction with the ligand VBL.

Figure S4. 2D interaction map (upper) and interacting residues (lower, highlighted in yellow) of CRO_E1 binding mode after MD simulation. A snapshot at 80 ns was selected as a representative structure.

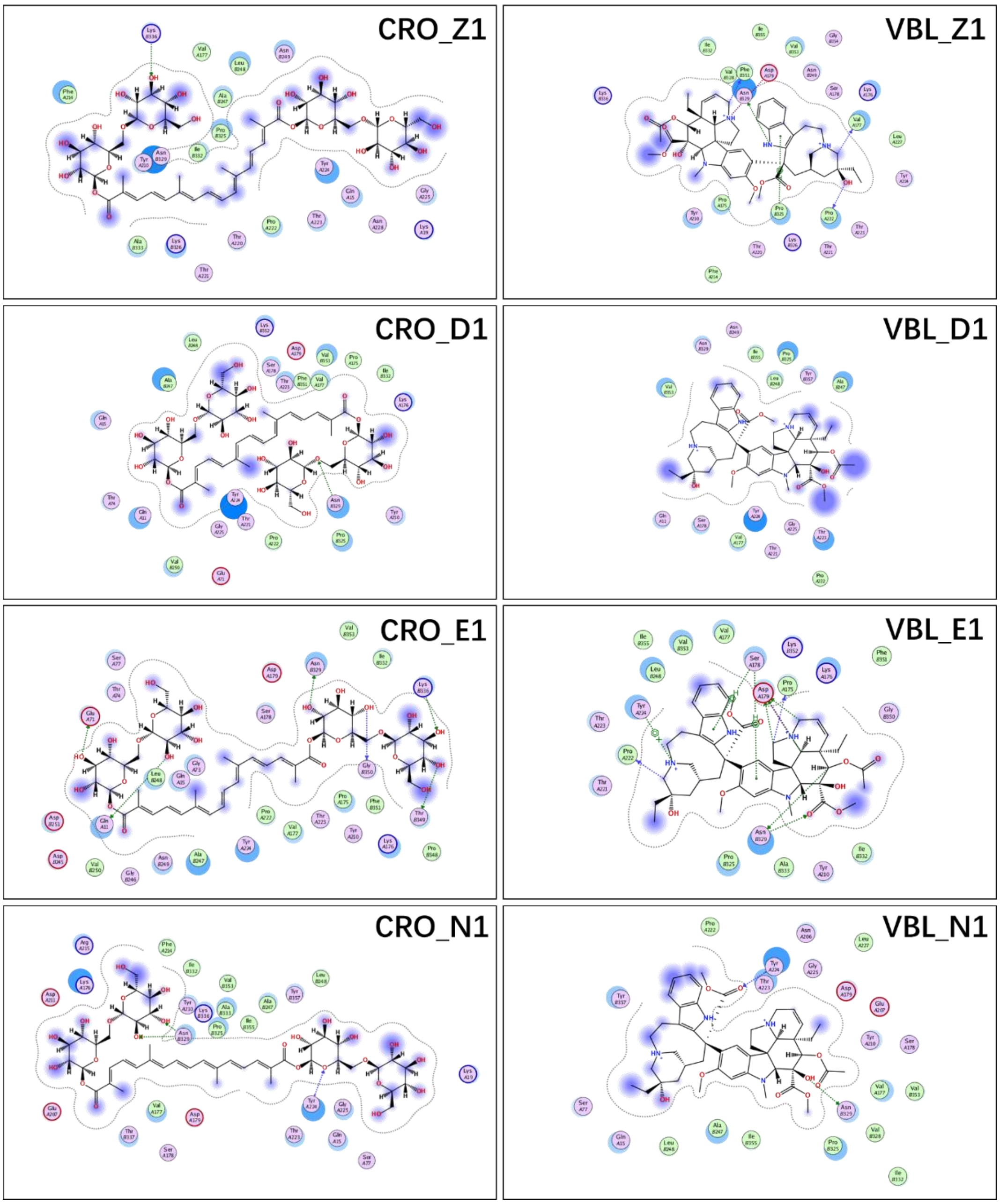

Figure S1.

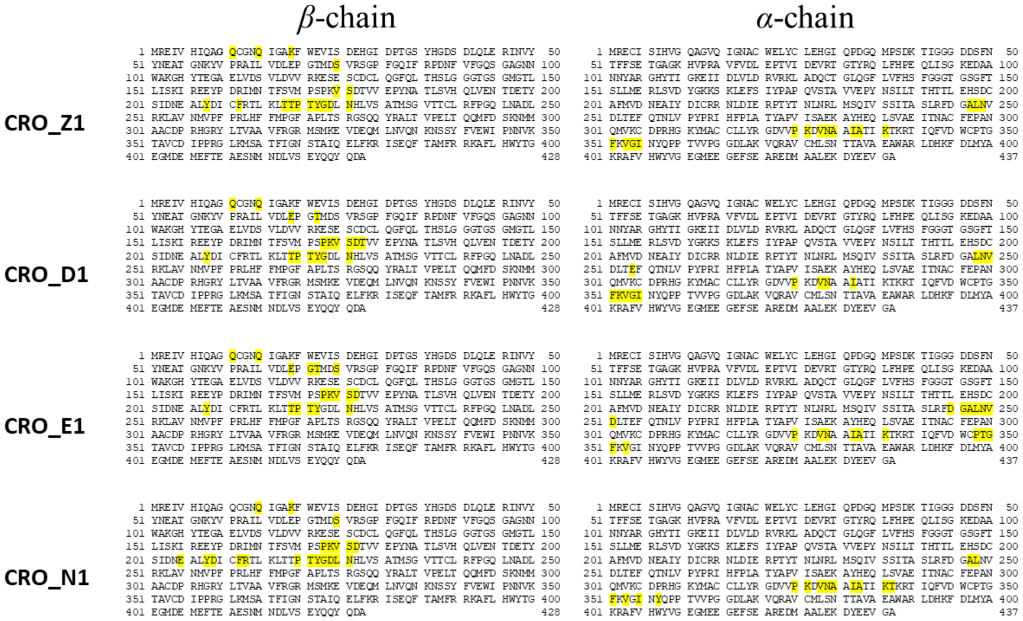

Figure S2.

β-chain | α-chain

VBL_Z1

```
  1 MREIV HIQAG QCGNQ IGAKF WEVIS DEHGI DPTGS YHGDS DLQLE RINVY  50
 51 YNEAT GNKYV PRAIL VDLEP GTMDS VRSGP FGQIF RPDNF VFGQS GAGNN 100
101 WAKGH YTEGA ELVDS VLDVV RKESE SCDCL QGFQL THSLG GGTGS GMGTL 150
151 LISKI REEYP DRIMN TFSVM PSPKV SDTVV EPYNA TLSVH QLVEN TDETY 200
201 SIDNE ALYDI CFRTL KLTTP TYGDL NHLVS ATMSG VTTCL RFPGQ LNADL 250
251 RKLAV NMVPF PRLHF FMPGF APLTS RGSQQ YRALT VPELT QQMFD SKNMM 300
301 AACDP RHGRY LTVAA VFRGR MSMKE VDEQM LNVQN KNSSY FVEWI PNNVK 350
351 TAVCD IPPRG LKMSA TFIGN STAIQ ELFKR ISEQF TAMFR RKAFL HWYTG 400
401 EGMDE MEFTE AESNM NDLVS EYQQY QDA                         428
```

```
  1 MRECI SIHVG QAGVQ IGNAC WELYC LEHGI QPDGQ MPSDK TIGGG DDSFN  50
 51 TFFSE TGAGK HVPRA VFVDL EPTVI DEVRT GTYRQ LFHPE QLISG KEDAA 100
101 NNYAR GHYTI GKEII DLVLD RVRKL ADQCT GLQGF LVFHS FGGGT GSGFT 150
151 SLLME RLSVD YGKKS KLEFS IYPAP QVSTA VVEPY NSILT THTTL EHSDC 200
201 AFMVD NEAIY DICRR NLDIE RPTYT NLNRL MSQIV SSITA SLRFD GALNV 250
251 DLTEF QTNLV PYPRI HFPLA TYAPV ISAEK AYHEQ LSVAE ITNAC FEPAN 300
301 QMVKC DPRHG KYMAC CLLYR GDVVP KDVNA AIATI KTKRT IQFVD WCPTG 350
351 FKVGI NYQPP TVVPG GDLAK VQRAV CMLSN TTAVA EAWAR LDHKF DLMYA 400
401 KRAFV HWYVG EGMEE GEFSE AREDM AALEK DYEEV GA                437
```

VBL_D1

```
  1 MREIV HIQAG QCGNQ IGAKF WEVIS DEHGI DPTGS YHGDS DLQLE RINVY  50
 51 YNEAT GNKYV PRAIL VDLEP GTMDS VRSGP FGQIF RPDNF VFGQS GAGNN 100
101 WAKGH YTEGA ELVDS VLDVV RKESE SCDCL QGFQL THSLG GGTGS GMGTL 150
151 LISKI REEYP DRIMN TFSVM PSPKV SDTVV EPYNA TLSVH QLVEN TDETY 200
201 SIDNE ALYDI CFRTL KLTTP TYGDL NHLVS ATMSG VTTCL RFPGQ LNADL 250
251 RKLAV NMVPF PRLHF FMPGF APLTS RGSQQ YRALT VPELT QQMFD SKNMM 300
301 AACDP RHGRY LTVAA VFRGR MSMKE VDEQM LNVQN KNSSY FVEWI PNNVK 350
351 TAVCD IPPRG LKMSA TFIGN STAIQ ELFKR ISEQF TAMFR RKAFL HWYTG 400
401 EGMDE MEFTE AESNM NDLVS EYQQY QDA                         428
```

```
  1 MRECI SIHVG QAGVQ IGNAC WELYC LEHGI QPDGQ MPSDK TIGGG DDSFN  50
 51 TFFSE TGAGK HVPRA VFVDL EPTVI DEVRT GTYRQ LFHPE QLISG KEDAA 100
101 NNYAR GHYTI GKEII DLVLD RVRKL ADQCT GLQGF LVFHS FGGGT GSGFT 150
151 SLLME RLSVD YGKKS KLEFS IYPAP QVSTA VVEPY NSILT THTTL EHSDC 200
201 AFMVD NEAIY DICRR NLDIE RPTYT NLNRL MSQIV SSITA SLRFD GALNV 250
251 DLTEF QTNLV PYPRI HFPLA TYAPV ISAEK AYHEQ LSVAE ITNAC FEPAN 300
301 QMVKC DPRHG KYMAC CLLYR GDVVP KDVNA AIATI KTKRT IQFVD WCPTG 350
351 FKVGI NYQPP TVVPG GDLAK VQRAV CMLSN TTAVA EAWAR LDHKF DLMYA 400
401 KRAFV HWYVG EGMEE GEFSE AREDM AALEK DYEEV GA                437
```

VBL_E1

```
  1 MREIV HIQAG QCGNQ IGAKF WEVIS DEHGI DPTGS YHGDS DLQLE RINVY  50
 51 YNEAT GNKYV PRAIL VDLEP GTMDS VRSGP FGQIF RPDNF VFGQS GAGNN 100
101 WAKGH YTEGA ELVDS VLDVV RKESE SCDCL QGFQL THSLG GGTGS GMGTL 150
151 LISKI REEYP DRIMN TFSVM PSPKV SDTVV EPYNA TLSVH QLVEN TDETY 200
201 SIDNE ALYDI CFRTL KLTTP TYGDL NHLVS ATMSG VTTCL RFPGQ LNADL 250
251 RKLAV NMVPF PRLHF FMPGF APLTS RGSQQ YRALT VPELT QQMFD SKNMM 300
301 AACDP RHGRY LTVAA VFRGR MSMKE VDEQM LNVQN KNSSY FVEWI PNNVK 350
351 TAVCD IPPRG LKMSA TFIGN STAIQ ELFKR ISEQF TAMFR RKAFL HWYTG 400
401 EGMDE MEFTE AESNM NDLVS EYQQY QDA                         428
```

```
  1 MRECI SIHVG QAGVQ IGNAC WELYC LEHGI QPDGQ MPSDK TIGGG DDSFN  50
 51 TFFSE TGAGK HVPRA VFVDL EPTVI DEVRT GTYRQ LFHPE QLISG KEDAA 100
101 NNYAR GHYTI GKEII DLVLD RVRKL ADQCT GLQGF LVFHS FGGGT GSGFT 150
151 SLLME RLSVD YGKKS KLEFS IYPAP QVSTA VVEPY NSILT THTTL EHSDC 200
201 AFMVD NEAIY DICRR NLDIE RPTYT NLNRL MSQIV SSITA SLRFD GALNV 250
251 DLTEF QTNLV PYPRI HFPLA TYAPV ISAEK AYHEQ LSVAE ITNAC FEPAN 300
301 QMVKC DPRHG KYMAC CLLYR GDVVP KDVNA AIATI KTKRT IQFVD WCPTG 350
351 FKVGI NYQPP TVVPG GDLAK VQRAV CMLSN TTAVA EAWAR LDHKF DLMYA 400
401 KRAFV HWYVG EGMEE GEFSE AREDM AALEK DYEEV GA                437
```

VBL_N1

```
  1 MREIV HIQAG QCGNQ IGAKF WEVIS DEHGI DPTGS YHGDS DLQLE RINVY  50
 51 YNEAT GNKYV PRAIL VDLEP GTMDS VRSGP FGQIF RPDNF VFGQS GAGNN 100
101 WAKGH YTEGA ELVDS VLDVV RKESE SCDCL QGFQL THSLG GGTGS GMGTL 150
151 LISKI REEYP DRIMN TFSVM PSPKV SDTVV EPYNA TLSVH QLVEN TDETY 200
201 SIDNE ALYDI CFRTL KLTTP TYGDL NHLVS ATMSG VTTCL RFPGQ LNADL 250
251 RKLAV NMVPF PRLHF FMPGF APLTS RGSQQ YRALT VPELT QQMFD SKNMM 300
301 AACDP RHGRY LTVAA VFRGR MSMKE VDEQM LNVQN KNSSY FVEWI PNNVK 350
351 TAVCD IPPRG LKMSA TFIGN STAIQ ELFKR ISEQF TAMFR RKAFL HWYTG 400
401 EGMDE MEFTE AESNM NDLVS EYQQY QDA                         428
```

```
  1 MRECI SIHVG QAGVQ IGNAC WELYC LEHGI QPDGQ MPSDK TIGGG DDSFN  50
 51 TFFSE TGAGK HVPRA VFVDL EPTVI DEVRT GTYRQ LFHPE QLISG KEDAA 100
101 NNYAR GHYTI GKEII DLVLD RVRKL ADQCT GLQGF LVFHS FGGGT GSGFT 150
151 SLLME RLSVD YGKKS KLEFS IYPAP QVSTA VVEPY NSILT THTTL EHSDC 200
201 AFMVD NEAIY DICRR NLDIE RPTYT NLNRL MSQIV SSITA SLRFD GALNV 250
251 DLTEF QTNLV PYPRI HFPLA TYAPV ISAEK AYHEQ LSVAE ITNAC FEPAN 300
301 QMVKC DPRHG KYMAC CLLYR GDVVP KDVNA AIATI KTKRT IQFVD WCPTG 350
351 FKVGI NYQPP TVVPG GDLAK VQRAV CMLSN TTAVA EAWAR LDHKF DLMYA 400
401 KRAFV HWYVG EGMEE GEFSE AREDM AALEK DYEEV GA                437
```

Figure S3.

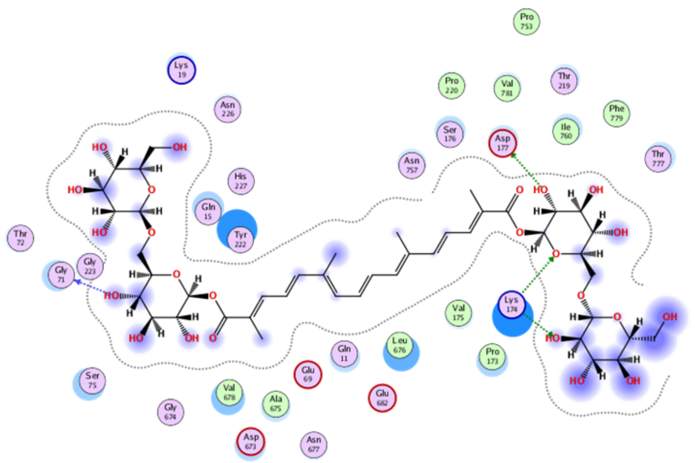

β-chain

```
  1 MREIV HIQAG QCGNQ IGAKF WEVIS DEHGI DPTGS YHGDS DLQLE RINVY  50
 51 YNEAT GNKYV PRAIL VDLEP GTMDS VRSGP FGQIF RPDNF VFGQS GAGNN 100
101 WAKGH YTEGA ELVDS VLDVV RKESE SCDCL QGFQL THSLG GGTGS GMGTL 150
151 LISKI REEYP DRIMN TFSVM PSPKV SDTVV EPYNA TLSVH QLVEN TDETY 200
201 SIDNE ALYDI CFRTL KLTTP TYGDL NHLVS ATMSG VTTCL RFPGQ LNADL 250
251 RKLAV NMVPF PRLHF FMPGF APLTS RGSQQ YRALT VPELT QQMFD SKNMM 300
301 AACDP RHGRY LTVAA VFRGR MSMKE VDEQM LNVQN KNSSY FVEWI PNNVK 350
351 TAVCD IPPRG LKMSA TFIGN STAIQ ELFKR ISEQF TAMFR RKAFL HWYTG 400
401 EGMDE MEFTE AESNM NDLVS EYQQY QDA                         428
```

α-chain

```
  1 MRECI SIHVG QAGVQ IGNAC WELYC LEHGI QPDGQ MPSDK TIGGG DDSFN  50
 51 TFFSE TGAGK HVPRA VFVDL EPTVI DEVRT GTYRQ LFHPE QLISG KEDAA 100
101 NNYAR GHYTI GKEII DLVLD RVRKL ADQCT GLQGF LVFHS FGGGT GSGFT 150
151 SLLME RLSVD YGKKS KLEFS IYPAP QVSTA VVEPY NSILT THTTL EHSDC 200
201 AFMVD NEAIY DICRR NLDIE RPTYT NLNRL MSQIV SSITA SLRFD GALNV 250
251 DLTEF QTNLV PYPRI HFPLA TYAPV ISAEK AYHEQ LSVAE ITNAC FEPAN 300
301 QMVKC DPRHG KYMAC CLLYR GDVVP KDVNA AIATI KTKRT IQFVD WCPTG 350
351 FKVGI NYQPP TVVPG GDLAK VQRAV CMLSN TTAVA EAWAR LDHKF DLMYA 400
401 KRAFV HWYVG EGMEE GEFSE AREDM AALEK DYEEV GA               437
```

Figure S4.
